# Microbial communities as biosensors for monitoring urban environments

**DOI:** 10.1111/1751-7915.12831

**Published:** 2017-08-15

**Authors:** Fangqiong Ling

**Affiliations:** ^1^ Department of Biological Engineering Massachusetts Institute of Technology 500 Technology Square Cambridge MA 02139 USA

## Abstract

The BE microbiome is a naturally embedded biosensor in urban infrastructure that can be used to monitor environmental quality and human activity. There are many potential opportunities for leveraging BE microbial communities to guide urban design and public health policy.

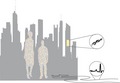

Advances in sequencing technology have fostered a better understanding of the microbes inhabiting the built environment (BE). Despite efforts to minimize bacterial and fungal biomass in the BE, we now know that BE air, surfaces and tap water harbour multitudes of microbes. BE microbial community composition is shaped by the surrounding environment (i.e. outdoor urban or natural environments) and by BE design choices, although the latter often take effect in unintended ways (Proctor and Hammes, 2015; Stephens, 2016). Additionally, prior work has demonstrated that microbes transferred to BE surfaces by humans can be forensically traced back to their sources (Lax *et al*., 2014; Franzosa *et al*., 2015). It is reasonable to ask, is there specific knowledge that can be translated into technologies that contribute to making urban environments more *safe*,* sustainable*,* inclusive* and *resilient*? (United Nations General Assembly). The BE microbiome is a naturally embedded biosensor in urban infrastructure that can be used to monitor environmental quality and human activity. There are many potential opportunities for leveraging BE microbial communities to guide urban design and public health policy.

It is important to provide access to *safe*, affordable public infrastructure. This target, which concerns delivery of basic public services, demands adequate urban infrastructure development. In many developed countries, much of the established urban infrastructure has reached its maximal life expectancy (American Society of Civil Engineers; Houlihan, 1994). In the United States, an estimated $ 4.6 trillion investment will be needed by 2025 (American Society of Civil Engineers) to update this crumbling infrastructure. In the case of water infrastructure, the countrywide distribution networks that deliver water from centralized water treatment facilities to households require the most attention. These distribution systems across the United States are dilapidated – for example, there are approximately 240,000 water main breaks per year (American Society of Civil Engineers) – and are increasingly vulnerable to the extreme weather events associated with climate change. Thus, the U.S. Environmental Protection Agency has determined innovation in water infrastructure monitoring and mitigation as a crucial research area (United States Environmental Protection Agency).

The microbiome of household water meters has been employed as a sensor device for citywide water quality surveillance (Hong *et al*., 2009; Ling *et al*., 2016). Compared to sampling tap water, sampling of water meters allows direct tracking of biofilms residing within the water distribution infrastructure. Biofilms are the majority of biomass in a water supply ecosystem. These water supply biofilms are known to harbour opportunistic pathogens such as *Mycobacterium avium* and are prone to detach into tap water during changes in flow regimes (Szewzyk *et al*., 2000; United States Environmental Protection Agency, 2002). My work in Urbana, Illinois established a baseline for this type of water‐system monitoring, identifying core populations in biofilm communities that were both abundant and prevalent (Hanski, 1982; Ling *et al*., 2016). The taxa identified would not have been detected by conventional biological monitoring tools (e.g. total coliform tests) (Ling *et al*., 2016). For this biofilm sensor system to be fully developed, more data are needed to define potential source populations under various compromised situations, such as pipe leakage, sewage intrusion and presence of dead‐end zones in a distribution network.

To face the challenge of global climate change, we must design more *sustainable* urban environments. Today, developed countries still discharge an unacceptable level of inadequately treated sewage into the environment. In the United States, 52.7% of rivers and streams and 79.5% of bays and estuaries are categorized as ‘impaired’. The leading cause of this impairment is cited as ‘pathogens’ (positive results from faecal indicator tests) (United States Environmental Protection Agency). Current regulation on environmental water quality relies on traditional faecal indicators (i.e. coliforms, *Escherichia coli*, or enterococci) (Freedman, 2004; Keller and Cavallaro, 2008; United States Environmental Protection Agency). These indicators are not very effective in tracing the source of pollution and guiding mitigation (McLellan and Eren, 2014). Host‐associated anaerobes, such as *Bacteroidales*, have been considered as a kind of alternative indicator due to higher host‐specificity. Various genetic assays based on the 16S rRNA genes or functional genes in *Bacteroidales* have been developed for this application, and showed high sensitivity and specificity. For example, using a hierarchical oligonucleotide primer extension (HOPE) assay on 16S rRNA genes of *Bacteroides* spp., Hong *et al*. (2010) showed that human, pig, cow and dog faeces can be correctly identified. Furthermore, these sources could be accurately tracked within contaminated bodies of water (81%). In another study, Verhougstraete *et al*. (2015) sampled across 64 rivers that drained 84% of Michigan's Lower Peninsula, and showed that the abundance of *Bacteroides thetaiotaomicron* alpha‐1‐6‐mannanase, a human‐specific marker, correlated strongly with the number of septic tanks in a watershed, and this correlation was not shown in standard coliform counts. Thus, culture‐independent methods provide much richer information than just a ‘yes’ or ‘no’ to the presence of pollution can help decision makers decide how to prioritize areas for the construction of new sewage treatment facilities, allowing for more targeted watershed restoration.

16S rRNA gene sequencing of whole microbial communities can provide a powerful source of environmental sensor data. Vast amounts of sequencing data enable the effective use of machine learning algorithms. Thus, source tracking expand from a small subset of host‐specific markers to thousands of phylotypes that differ in relative abundance across samples, allowing for more sensitive assays (McLellan and Eren, 2014). Oligotyping, a computational method that makes use of highly variable nucleotide positions in 16S sequences, can distinguish closely related organisms that would normally be classified into one taxon (Eren *et al*., 2013). Oligotyping has led to the identification of human faecal indicators from multiple taxonomic groups (Koskey *et al*., 2014; Fisher *et al*., 2014). Human‐specific taxa were found not only in populations of *Bacteroidetes*, but also in the bacterial phylum *Firmicutes*, including oligotypes under the genera *Blautia*,* Coprococcus*,* Dorea*,* Faecalibacterium* and *Roseburia* (Fisher *et al*., 2014). In addition, community profiling of pipes transmitting sewage from households to wastewater treatment facilities has shown that *Arcobacter* species are abundant and prevalent, suggesting that using community composition in pollution source tracking has the potential to detect signatures from urban water infrastructure (Fisher *et al*., 2015).

Aside from sensing infrastructure safety and human impacts on the environment, the microbiome may even serve as an indicator for the *inclusiveness* of urban spaces. It has been reported that humans influence indoor environment microbiome by direct contact and emission of bioaerosols (Meadow *et al*., 2014; Adams *et al*., 2016; Stephens, 2016). For example, Kembel *et al*. found that the physical connectedness of building spaces correlated strongly with similarity in microbial community composition. Furthermore, certain taxa were indicative of whether an office is centrally located and hence more occupied (Kembel *et al*., 2014). Microbial communities on dust particles are partially sourced from human skin (Kembel *et al*., 2014; Meadow *et al*., 2015), and might be useful as a non‐intrusive data source to indicate the accessibility of an urban space from its use frequency. This could provide feedback for architectural design.

These are just a few examples of where the BE microbiome can be used as a biosensor. The microbiome is exquisitely sensitive and can detect events or conditions that are difficult or impossible to assess with traditional indicators. The physical carriers of the microbiome are easy to access (for example, water, surface residues or dust), which would allow passive sampling. These characteristics make microbiome sensors a valuable addition to current management and design toolkits. In bridging the scientific understandings to real‐world applications, however, the complexities of ecosystems in natural or built environments need to be considered. For instance, the decay of *B. ovatus* over time was shown to be strongly affected by UV irradiation. A *B. ovatus* contamination event during the day (i.e. when exposed to full‐spectrum UV irradiation) would decay more rapidly than a similar event in the evening (Dong *et al*., 2014). The BE microbiome field is nascent and developing rapidly and is likely to inspire new technologies that facilitate sustainable development goals.
